# A competitive and reversible deactivation approach to catalysis-based quantitative assays

**DOI:** 10.1038/ncomms10691

**Published:** 2016-02-19

**Authors:** Kazunori Koide, Matthew P. Tracey, Xiaodong Bu, Junyong Jo, Michael J. Williams, Christopher J. Welch

**Affiliations:** 1 Department of Chemistry, University of Pittsburgh, 219 Parkman Avenue, Pittsburgh, Pennsylvania 15260, USA; 2 Process and Analytical Chemistry, Merck Research Laboratories, 126 East Lincoln Avenue, Rahway, New Jersey 07065, USA

## Abstract

Catalysis-based signal amplification makes optical assays highly sensitive and widely useful in chemical and biochemical research. However, assays must be fine-tuned to avoid signal saturation, substrate depletion and nonlinear performance. Furthermore, once stopped, such assays cannot be restarted, limiting the dynamic range to two orders of magnitude with respect to analyte concentrations. In addition, abundant analytes are difficult to quantify under catalytic conditions due to rapid signal saturation. Herein, we report an approach in which a catalytic reaction competes with a concomitant inactivation of the catalyst or consumption of a reagent required for signal generation. As such, signal generation proceeds for a limited time, then autonomously and reversibly stalls. In two catalysis-based assays, we demonstrate restarting autonomously stalled reactions, enabling accurate measurement over five orders of magnitude, including analyte levels above substrate concentration. This indicates that the dynamic range of catalysis-based assays can be significantly broadened through competitive and reversible deactivation.

## Abstract (web summary)

Assays for catalytic systems—particularly ones with simple colorimetric readouts—are useful for the rapid evaluation of performance. Here, the authors report an assay based on a concurrent colour-forming reaction working across a wide range that can be stopped to allow measurements and subsequently restarted.

## Graphical abstract

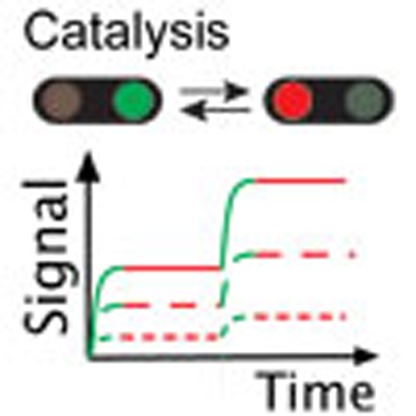

Development of optical assays for facile quantification of trace analytes is an ever-expanding field. Target analytes range from trace metals^1^ and biological signalling agents^2^,^3^ to chemical weapons^4^. Tailored chemosensors interact specifically with an analyte to produce an optically decoded signal, which can manifest itself as a wavelength shift or intensity change in either absorbance or emission. These signals are measured by simple instrumentation, such as a plate reader, or visualized with the naked eye.

Quantitative optical assays exploit either a catalytic or a non-catalytic reaction. Non-catalytic assays rely on a single turnover from the analyte for a chemical conversion or a reversible binding and have the benefit of time-independence; in other words, the signal does not change over time once the reaction or binding event is complete. A major drawback of these systems is the higher limits of quantification due to this limited turnover, rendering these non-catalytic assays undesirable for detection of trace analytes. A more sensitive approach for trace analytes uses catalysis-based assays, where the substrate continues to react over time, amplifying signals.

The continuity of catalysis-based signal amplification presents some practical challenges to assay development. In metal catalysis-based assays, once the metal has entered into the catalytic cycle, the resulting fluorescence signal is dependent on the concentration of the analyte as well as the time elapsed, with the reaction continuing until the fluorogenic substrate is consumed. In enzyme and enzyme-linked immunosorbent assays^5^, the reaction continues until the substrate is consumed or a terminating reagent is added^6^. In either case, if an analyte is abundant, the assay substrate will be rapidly consumed, preventing accurate quantitation. In addition, if a reaction with a low concentration of analyte is allowed to continue unchecked, the signal can increase to the point where the detector becomes saturated, again preventing accurate quantification. Finally, when a catalysis-based assay is externally stopped, it cannot be restarted^6^ and premature termination requires the assay to be repeated to obtain quantitative data. As such, a significant drawback associated with catalysis-based assays is the far narrower dynamic range (one to two orders of magnitude) compared with more labour-intensive methods, such as inductively coupled plasma mass spectrometry (ICP-MS), which has a detection range is up to five orders of magnitude. New methodologies that overcome these limitations to enable controlled activity of catalytic assays would be broadly useful in chemical and biochemical research.

Herein, we present a new approach to catalysis-based assays in which a catalytic chromogenic reaction competes with the deactivation of the catalyst or depletion of an essential reagent. Under these conditions, a signal-producing reaction proceeds for a limited time, then autonomously stalls, but can be reactivated by reagent addition, generating a graph reminiscent of a staircase function in mathematics. This approach is exemplified by both a new colorimetric method for quantifying palladium (Pd) and a horseradish peroxidase assay system. In the analysis of Pd, multiple cycles of reaction stalling and restarting allow accurate measurement with a detection range of over five orders of magnitude. Moreover, analyte levels significantly above the substrate concentration can be quantified.

## Results

### Resorufin allyl ether as a chemodosimeter for palladium

**Figure 1:**
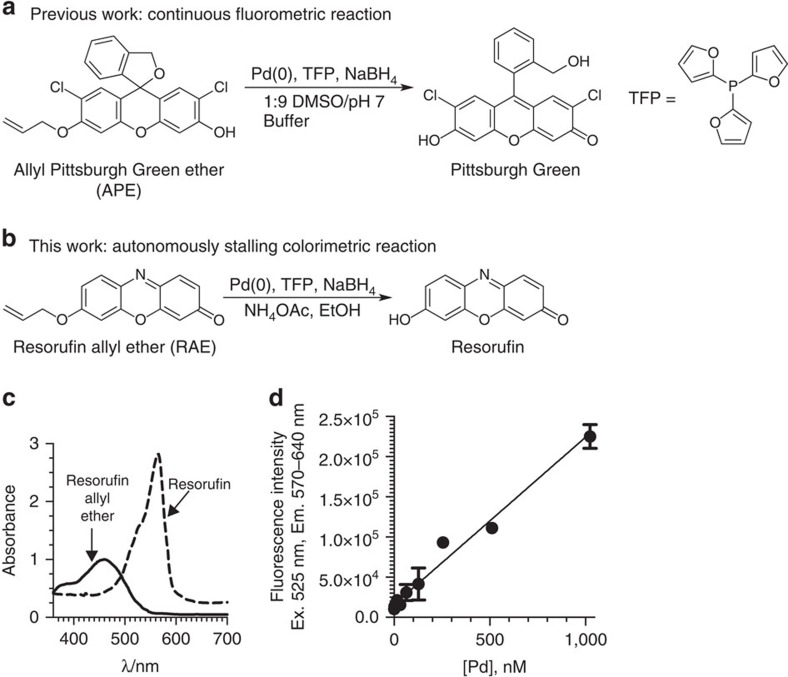
Palladium chemodosimeters based on change in optical properties. (**a**) Structures of previously developed fluorogenic chemodosimeter APE and its conversion to Pittsburgh Green. (**b**) Chromogenic chemodosimeter RAE and its conversion to resorufin. (**c**) Absorption spectra of resorufin and RAE in 800 mM NH_4_OAc in EtOH. The data are normalized to 20 μM of each compound. (**d**) Correlation between Pd concentrations and fluorescence signal using RAE. *r*^2^=0.97, *y*=(208±7.38)*x*+(15,910±2,765). Conditions: 29 μM RAE, 0, 8, 16, 32, 64, 128, 256, 512, 1,024 nM Pd(II). 50 mM NaBH_4_, 200 μM TFP, 800 mM NH_4_OAc, EtOH, 24 °C, 60 min; *n*=3.

We previously reported a fluorescence method for quantifying Pd in pharmaceuticals based on the Pd-catalysed fluorogenic conversion of allyl Pittsburgh Green ether (APE) to Pittsburgh Green (Fig. 1a)^7^,^8^,^9^,^10^. Although this method showed excellent sensitivity and an ability to accurately quantify low-level Pd in real-world samples, we realized that a colorimetric version of the assay could allow even simpler, instrument-free access to low-level Pd measurements, a goal previously attempted by several other researchers with limited success^11^,^12^.

Investigation of a number of candidate chromogenic substrates led to the preparation of yellow-coloured resorufin allyl ether (RAE) in one step in 85% yield from commercially available purple-coloured resorufin (Fig. 1b,c, Supplementary Figs 13 and 14). Attempts at Pd-catalysed deallylation of RAE using the optimized conditions for APE (tris(2-furyl)phosphine (TFP)), NaBH_4_, dimethyl sulphoxide (DMSO)/1.23 M phosphate pH 7 buffer (1:9)) were unsuccessful. However, screening a variety of commercially available phosphines and additives (Supplementary Table S1 and Supplementary Figs 1–3) led to the identification of suitable conditions for carrying out the transformation. Optimized conditions for the Pd-dependent deallylation of RAE used TFP, NH_4_OAc and NaBH_4_ in an EtOH solvent. Further optimization of RAE as a substrate can be found in the Supplementary Methods and Supplementary Figs 2–4.

We found that RAE was selectively responsive to Pd over other metals tested (Ag, Au, Cd, Co, Cr, Fe, Hg, Mn, Ni. Pt, Rh, Ru, Zn, Sr, Ir, Cu; Supplementary Fig. 4a,b) and could detect Pd without interference from these metals, with the exception of Hg, where a small level of interference was observed (Supplementary Fig. 4c). When selectivity was tested by absorbance, higher values were observed in the presence of Au, Ag, and Hg owing to turbidity of the solution, although fluorescence measurement revealed that these were merely false positives (that is, these metals did not convert RAE to resorufin; Supplementary Fig. 4a,b). When exposed to Pd, the fluorescence signal increased linearly with respect to Pd concentration (Fig. 1d), indicating a first-order relationship suitable for convenient quantification.

**Figure 2:**
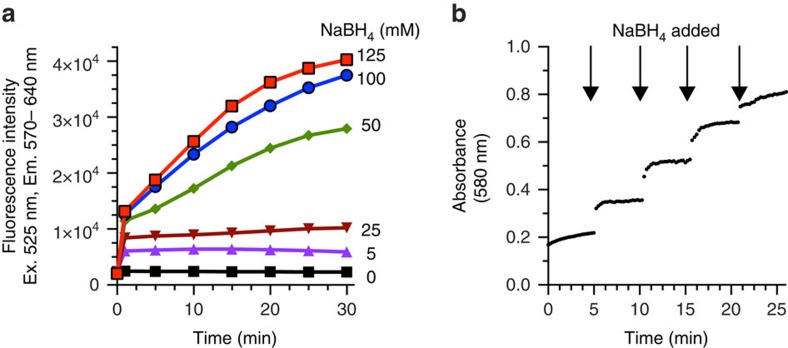
Influence of NaBH_4_ on deallylation of RAE. (**a**) Reaction lifetime dictated by NaBH_4_ concentration. Conditions: 29 μM RAE, 10 p.p.b. Pd(II), 200 μM TFP, 800 mM NH_4_OAc, 0–125 mM NaBH_4_, EtOH, 25 °C. (**b**) Stalled deallylation reaction can be restarted by NaBH_4_ addition. Conditions: 29 μM RAE, 0.3 p.p.m. Pd(II), 200 μM TFP, 800 mM NH_4_OAc, 0. 0.6, 1.2, 1.8, 2.4 mM NaBH_4_, added as 2.5 M aliquots at indicated time points.

The Pd-catalysed deallylation of APE in phosphate buffer was more effective in the presence of NaBH_4_, which reduces Pd(II) and Pd(IV) to catalytically active Pd(0), but did not require this reducing agent as a critical component^9^. In contrast, Pd(II) species did not catalyse the deallylation of RAE in NH_4_OAc-containing EtOH without the reducing agent, with the amount of NaBH_4_ dictating the duration of reaction (Fig. 2a). This novel NaBH_4_-dependence boded well with our aim at competitively and reversibly deactivating catalysis-based assays, as detailed below. Lower concentrations of NaBH_4_, ranging from 5–25 mM, led to stalling of the colour-forming reaction within 30 s, presumably because of rapid consumption of the reductant, NaBH_4_, combined with ongoing air-oxidation of catalytically active Pd(0) to higher valent, inactive Pd species. In contrast, NaBH_4_ concentrations in excess of 50 mM allowed the reaction to continue for several minutes. Importantly, the addition of more NaBH_4_ could restart a stalled deallylation reaction (Fig. 2b), affording a convenient way to trigger signal generation on demand.

Subsequently, we sought to gain insights into the reaction stalling to rationally expand this developing methodology. The Pd-catalysed deallylation of APE stalled in the presence of NH_4_OAc but continued in a phosphate buffer (Supplementary Fig. 6). With 200, 400, 600 and 800 mM NH_4_OAc followed by pH adjustment, the reactions stalled nearly at the same time (Supplementary Fig. 7). The Pd-catalysed deallylation reaction of RAE under a nitrogen atmosphere were found to stall more slowly than those carried out in open air (Supplementary Fig. 8), suggesting that aerobic oxidation of Pd(0) to higher order Pd species may account for the observed reaction stalling.

### Demonstration of stop-and-go methodology with RAE

In an effort to develop a simple, user-friendly colorimetric Pd quantification assay, we prepared a reagent cocktail combining all reaction components except NaBH_4_ in a single solution. This cocktail, which is stable for over 2 weeks when stored at 5 °C, can be dispensed as needed, simplifying application of the colorimetric method. The addition of either 20 μl of a solution or 2–5 mg of a solid sample containing trace Pd to 1 ml of the reaction cocktail, followed by the addition of a NaBH_4_ solution, generated colour and fluorescence within 1 min. The colour intensity was linearly correlated with Pd concentration, and the dynamic range and reaction time of the assay were tailored by adjusting the NaBH_4_ concentration.

**Figure 3:**
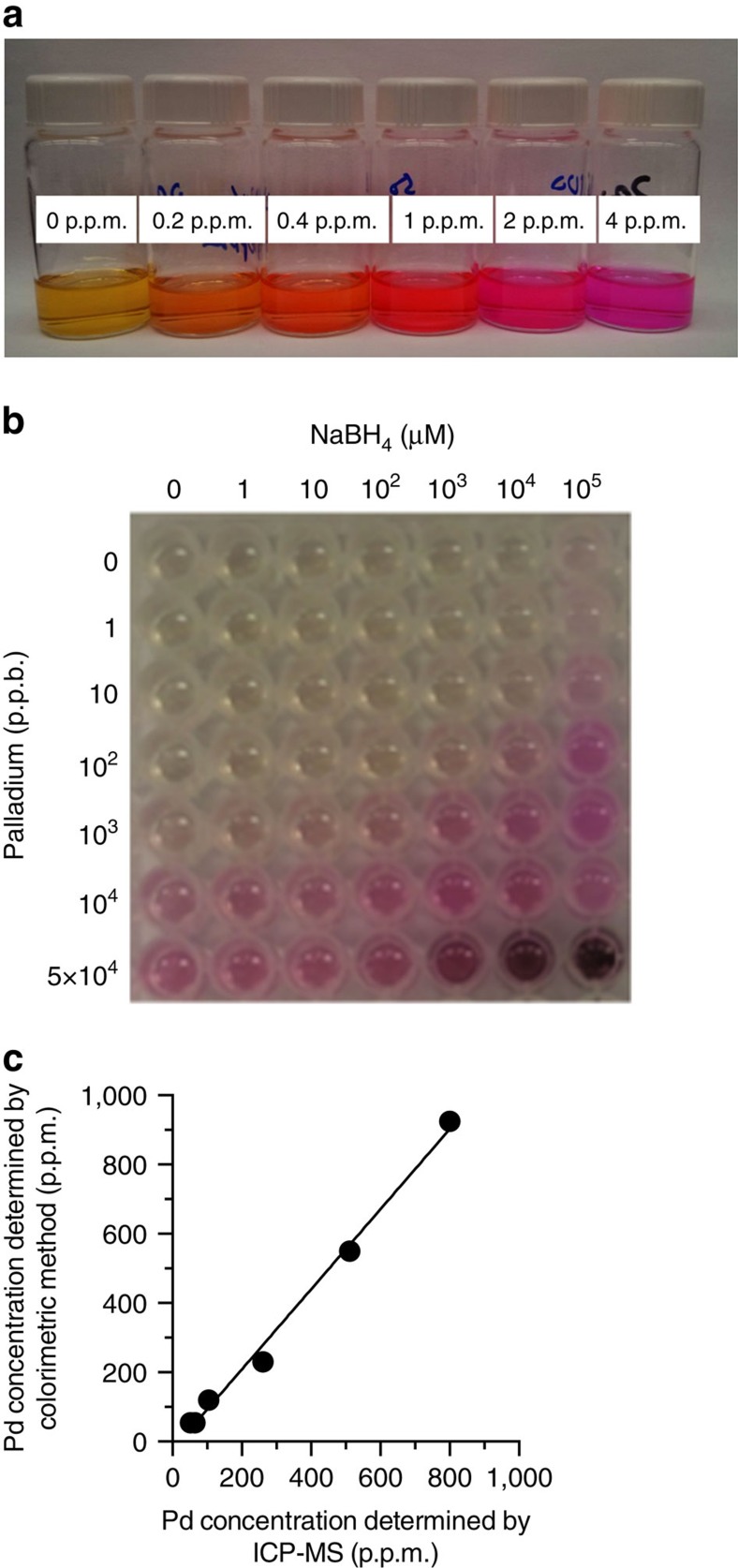
Visual quantification of palladium with RAE. (**a**) The appearance of distinguishable colour correlating to Pd concentration occurs in less than 1 min using a set of Pd standards. Conditions: 29 μM RAE, 0–4.0 p.p.m. Pd, 200 μM TFP, 800 mM NH_4_OAc, 1.0 mM NaBH_4_, EtOH, 25 °C. (**b**) Colorimetric plate showing dependence of colour formation on Pd and NaBH_4_ concentration; 29 μM RAE, 200 μM TFP, 0–50 p.p.m. Pd, 0–100 mM NaBH_4_, 800 mM NH_4_OAc 25 °C, EtOH, 10 min, *n*=3. (**c**) Conditions: colorimetric analysis as reported in Fig. 4a; ICP-MS analysis as reported in the ‘Methods' section.

The power of this method is shown in Fig. 3. Known concentrations of Pd afford widely different colours with a single concentration of NaBH_4_, with the colour persisting for 24 h (Fig. 3a). If a sample contains 1 p.p.b. Pd, then 100 mM NaBH_4_ is required to observe a colour change (Fig. 3b). If a sample contains 10 p.p.m. Pd, then no NaBH_4_ is added to observe a colour change. Thus, Pd concentrations ranging from 1 p.p.b. to 10 p.p.m. (five orders of magnitude) can be distinguished in one reaction solution with NaBH_4_ titration. Alternatively, a user may prepare multiple wells with variable NaBH_4_ amounts and count a number of coloured wells to estimate the Pd concentrations.

To confirm that the stop-and-go assay approach is providing quantitative data, we analysed real-world samples. We first tested intermediates used in the preparation of active pharmaceutical ingredients. In pharmaceutical synthesis, reactions may leave behind residual Pd in the products, which is often difficult to remove^13^. Various samples were tested from active projects in the Process and Analytical Chemistry Department at Merck Research Laboratories in which residual Pd removal has proven difficult. Quantification of Pd was initially performed by ICP-MS followed by analysis using RAE. Compared with the ICP-MS analysis, the stop-and-go approach with RAE provided accuracy from 70 to 120%, with residual Pd concentrations ranging from 62 to 800 p.p.m. (Fig. 3c). These results were satisfactory for this assay approach to be used for screening dozens of routine Pd remediation protocols.

Microscale screening of process adsorbents is often used to identify resins or activated carbons that can be used for selective adsorption of metal impurities in pharmaceutical process research and development^14^,^15^. Traditionally, this approach requires close coordination with ICP-MS specialists to allow for quick turnaround time. However, often because of instrument calibration, the vast number of samples, and preparation time, this can be time consuming. As such, the pharmaceutical industry has been interested in a faster technology for trace metal analysis^16^.

**Figure 4:**
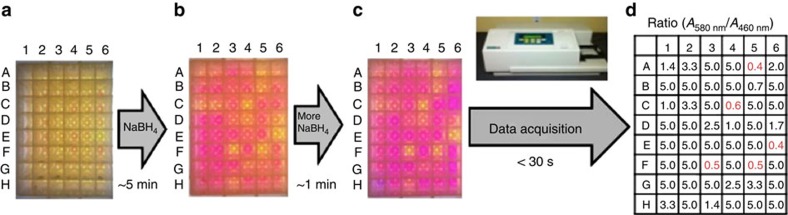
Case study of a streamlined process combining adsorbent screening for Pd removal with high-throughput colorimetric Pd detection. (**a**) Screening kits containing 48 commercial adsorbents^15^ are exposed to a solution of a Pd-containing intermediate. (**b**) Aliquots from screening kits are evaluated for Pd content using the colorimetric method, as described in protocol. (**c**) Finding the best potential hits visually by adding more NaBH_4_. (**d**) High-throughput mapping of relative Pd concentration by measurement of ultraviolet–visible 570 nm/460 nm using ultraviolet–visible plate reader.

The application of the colorimetric method enables rapid determination of Pd concentrations ‘on the spot', in the same laboratory where the process development studies are being carried out. Figure 4 shows the results of a high throughput screen of Pd impurity remediation treatments of a pharmaceutical intermediate with 48 metal-scavenging adsorbents, using the stop-and-go assay with RAE to visualize relative Pd levels. An aliquot from each well is treated with the reaction cocktail (Fig. 4a), then with NaBH_4_. In less than 5 min, gross differences in Pd concentration are readily apparent to the naked eye by distinguishable colours (Fig. 4b). At this point, the reaction had stalled, and too many hits were identified. Accordingly, more NaBH_4_ was added to restart the reaction, accentuating the differences between wells and enabling rapid determination of the potential most-effective treatments for residual Pd remediation (Fig. 4c). A high-throughput mapping of relative Pd concentration was obtained by plotting the ratio of absorbance at 580 and 460 nm using a ultraviolet–visible plate reader (read time for 48 samples <30 s; Fig. 4d). These results quantitatively confirm the most effective Pd removal treatments to be wells A5, C4, E6, F3 and F5. Spot-checking several adsorbent treatment samples using conventional ICP-MS showed a good correlation with the colorimetric method, with the selection of the most-effective adsorbent treatments (A5, E6) being identical in both cases. These results demonstrate the utility of a stop-and-go approach in trace metal quantification, providing an important advance for process chemists dealing with remediation of Pd impurity problems using point-of-use high-throughput analysis.

We previously applied APE for quantifying Pd in ore samples without requiring acidic sample digestion^17^, a significant improvement over standard analytical methods such as ICP-MS, but still requiring the use of a blue light source to check fluorescence intensity. This requirement associated with the fluorometric method was not ideal for turbid samples such as ores. Therefore, we applied our colorimetric method for more convenient visualization. We found that the reaction was stalled before effective Pd extraction from the ores, leading to a need to add a large excess of NaBH_4_. However, a 1-min pre-incubation in a DMSO solution of TFP with sonication, followed by the addition of RAE, NH_4_OAc, EtOH and NaBH_4_ afforded subpar semi-quantitative data. A second addition of NaBH_4_ after the reaction had stalled provided good colorimetric agreement with previous semi-quantitative analysis^17^ (Supplementary Fig. 9) within 20 min.

Trace Pd is also a significant concern in materials science^18^,^19^. Our colorimetric method could be used to successfully detect trace Pd in polymer materials, as shown in Supplementary Figs 10–12, Supplementary Table 2 and Supplementary Notes 1 and 2.

### Competitive and reversible deactivation in enzymatic assay

**Figure 5:**
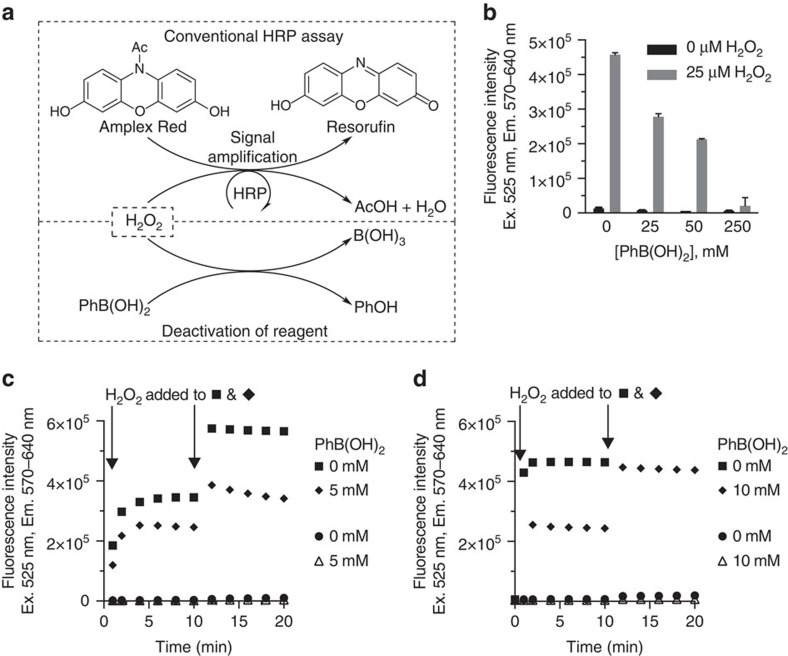
Stop-and-go approach in a horseradish peroxidase system. (**a**) Conversion of Amplex Red to resorufin. (**b**) Effect of PhB(OH)_2_ on horseradish peroxidase assay. Conditions: 50 μM Amplex Red, 0.1 U ml^−1^ horseradish peroxidase, 25 μM H_2_O_2_, 0, 25, 50, 250 mM PhB(OH)_2_, PBS pH 7.4. (**c**) Restarting a stopped enzymatic reaction in the presence of an inhibitor. Conditions: 50 μM Amplex Red, 0.05 U ml^−1^ horseradish peroxidase, 0 μM H_2_O_2_ (0–20 min) for the circle and triangle. For others, 10 μM H_2_O_2_ at 0 min, 20 μM H_2_O_2_ at 10 min, PBS pH 7.4. (**d**) Restarting a stopped enzymatic reaction in the presence of an inhibitor with uninhibited saturation. Conditions: 50 μM Amplex Red, 1 U ml^−1^ horseradish peroxidase, 0 μM H_2_O_2_ (0–20 min) for the circle and triangle. For others, 10 μM H_2_O_2_ at 0 min, 30 μM H_2_O_2_ at 10 min, PBS pH 7.4. After the addition of H_2_O_2_ at 10 min, the PhB(OH)_2_-free sample (square) showed a signal above the upper limit of the instrument (above 2 × 10^6^ units).

Horseradish peroxidase (HRP) is a common enzyme for detection and quantification in biological assays^20^,^21^. This enzyme catalytically converts Amplex Red (10-acetyl-3,7-dihydroxyphenoxazine) and H_2_O_2_ to resorufin, acetic acid and H_2_O. (Fig. 5a)^22^. The reaction continues indefinitely until either Amplex Red or H_2_O_2_ is consumed, at which point, signal generation is stopped.

To illustrate the discontinuous catalysis approach in a different assay, we designed a system in which PhB(OH)_2_ would competitively reduce H_2_O_2_ (Fig. 5a ‘Deactivation of reagent') while the H_2_O_2_-mediated oxidation of Amplex Red occurs (‘Conventional HRP assay'). Figure 5b shows that PhB(OH)_2_ was able to do so in a concentration-dependent manner, affording lower signals. Reactions halted by consumption of H_2_O_2_ could be restarted by an addition of a fresh aliquot of H_2_O_2_ (Fig. 5c). With a further addition of H_2_O_2_, signal saturation occurred (Fig. 5d). With the inclusion of the competitive scavenger, PhB(OH)_2_ to remove H_2_O_2_ from the system, the discontinuous catalysis alleviated the problem of overshooting signals, as well as allowed us to restart the reaction without problematic increases in fluorescence (Fig. 5d). Although the protocol has not been fully optimized in an HRP system, these data indicate a great potential for the applications of discontinuous catalysis in other enzyme assays.

## Discussion

**Figure 6:**
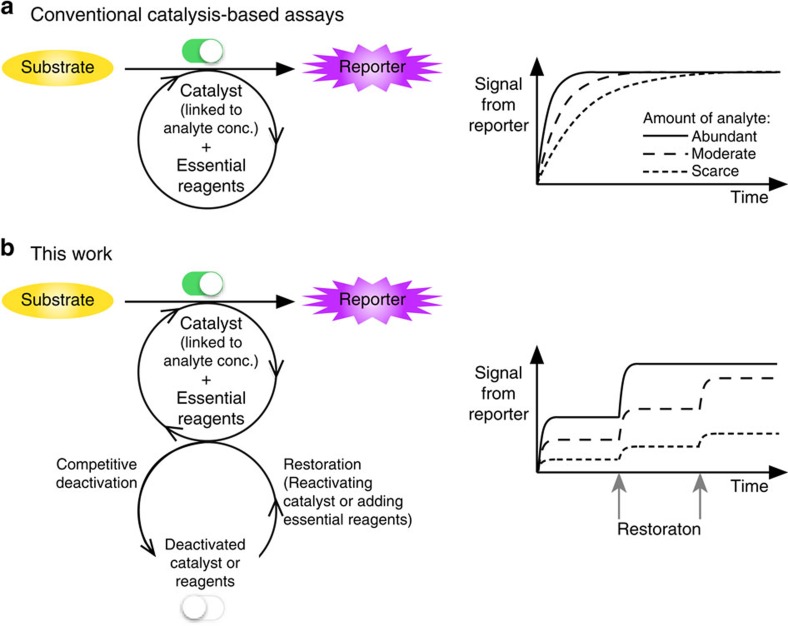
Continuous reaction and competitively and reversibly deactivated reaction. (**a**) Widely used catalytic assays continuously convert a substrate to a reporter molecule. (**b**) This work presents a stop-and-go paradigm, in which there is a competition between the catalytic reaction and autonomous deactivation of the catalyst (that is, analyte) or the essential reagent. The addition of an activator or a reactant restores the system.

We have developed a competitive and reversible deactivation approach for catalytic quantification assays (Fig. 6). Conversion of RAE to resorufin via a Pd-catalysed Tsuji–Trost reaction is autonomously stalled by the oxidation of reactive Pd(0) to non-reactive species. The addition of NaBH_4_ as a reducing agent is able to restart the reaction, enabling accurate measurements over five orders of magnitude. Notably, even in cases where the amount of Pd far exceeds the amount of RAE, the data remain quantitative. We have also demonstrated the utility of the same concept in a widely used HRP assay system, where competitive destruction of H_2_O_2_ by PhB(OH)_2_ leads to reaction stalling, broadening the dynamic range of the assay. These approaches should be compatible with automation and may find further applicable arenas to broaden the dynamic ranges of catalysis-based assays.

## Methods

### Ultraviolet–visible spectroscopy

The ultraviolet–visible spectra of RAE and resorufin solutions were acquired using a diode array spectrophotometer (Agilent Technologies, Santa Clara, CA) in a quartz cuvette. Other absorbance measurements were recorded in either a 96-well plate using a Modulus II Microplate Multimode reader (Promega, Madison, WI) measuring absorbance at 560 nm or in a clear, round bottom 96-well plates on a Spectra Max M5 spectrometer (Molecular Devices, Sunnyvale, CA) under the control of a Windows-based PC running software pro V5. The samples were analysed at *λ*=580 nm for the resorufin, and at *λ*=525 nm for RAE.

### Fluorescence measurement

Fluorescence measurements were read on a Modulus II Microplate Multimode Reader (excitation 525 nm, emission 580–640 nm) or using a HoribaMax Fluorometer (excitation 578 nm, emission 350–700 nm).

### Metal analysis by ICP-MS

The samples were either diluted or suspended directly in concentrated nitric acid or evaporated with a rotary evaporator first and then re-dissolved in concentrated nitric acid for ICP-MS analysis. Depending on the concentration range of the element, either a Perkin-Elmer Elan 6000 quadrupole ICP-MS spectrometer (Perkin-Elmer, Norwalk, CT) or a Thermo Finnigan Element 2 high-resolution ICP-MS spectrometer (Finnigan, Bremen, Germany) was used for the analysis.

### General protocol for deallylation of RAE

A reaction cocktail was prepared by mixing 800 mM NH_4_OAc in EtOH (10 ml) with 800 μM RAE in EtOH (400 μl) and 3 mM TFP in DMSO, with 250 p.p.m. BHT (800 μl). The reaction cocktail (1 ml) was added to individual 2-ml Eppendorf tubes. To half of the samples was added 5% TraceMetal HNO_3_ (20 μl) as a control. To the other half of the samples was added a Pd^2+^ solution in 5% TraceMetal HNO_3_ (20 μl). To all the samples was added NaBH_4_ in 10 N NaOH (20 μl). The samples were mixed and transferred (200 μl) to a 96-well black fluorescence well plate. Fluorescence (excitation 525 nm, emission 570–640 nm) was measured every 2 min for 60 min using a Modulus II Microplate Multimode Reader.

## Additional information

**How to cite this article:** Koide, K. *et al.* A competitive and reversible deactivation approach to catalysis-based quantitative assays. *Nat. Commun.* 7:10691 doi: 10.1038/ncomms10691 (2016).

## Supplementary Material

Supplementary Information Supplementary Figures 1-14, Supplementary Tables 1-2, Supplementary Notes 1-2, Supplementary Methods and Supplementary Reference
