# Supplementary material for: A competitive and reversible deactivation approach to catalysis-based quantitative assays
Source: Nat Commun. 2016 Feb 19;7:10691. doi: 10.1038/ncomms10691 (PMC4762883; doi:10.1038/ncomms10691)
Supplement: Supplementary Information — Supplementary Figures 1-14, Supplementary Tables 1-2, Supplementary Notes 1-2, Supplementary Methods and Supplementary Reference [file ncomms10691-s1.pdf]

Supplementary Information

Supplementary Figures

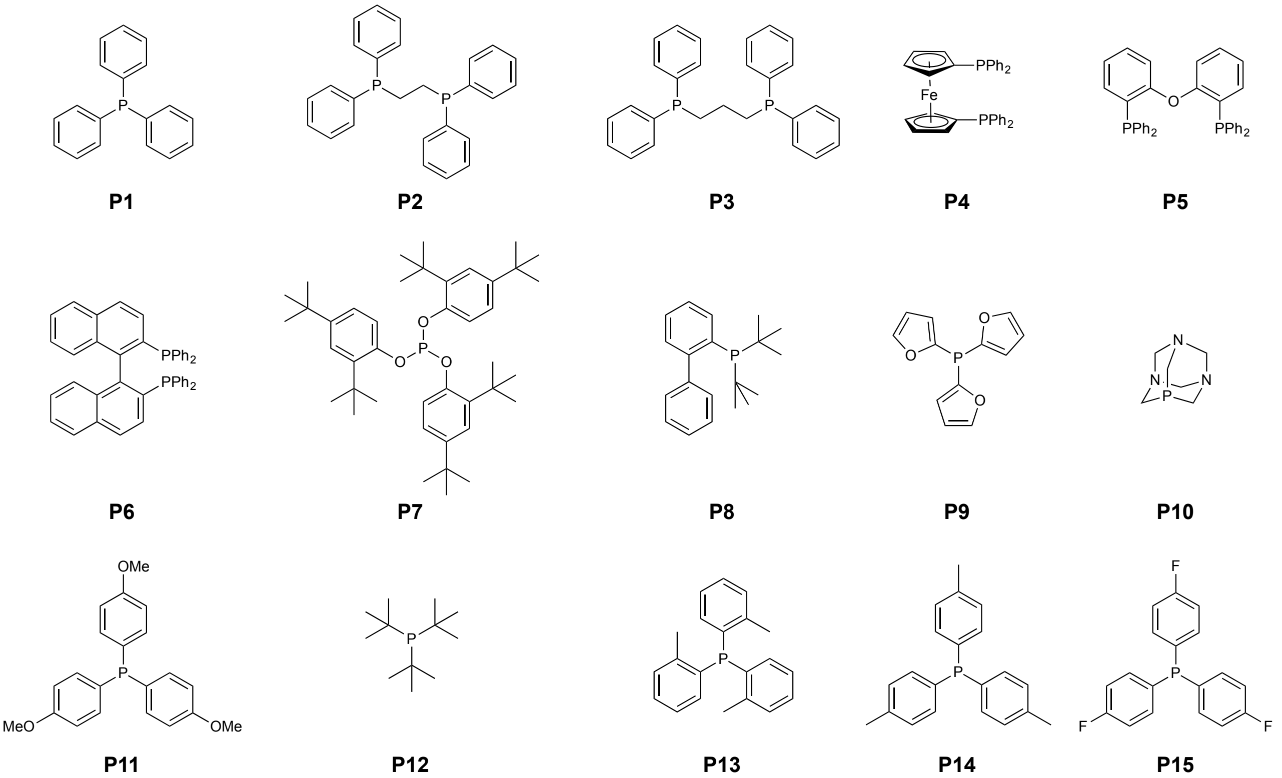

Supplementary Figure 1. Phosphines screened for deallylation of RAE to resorufin.

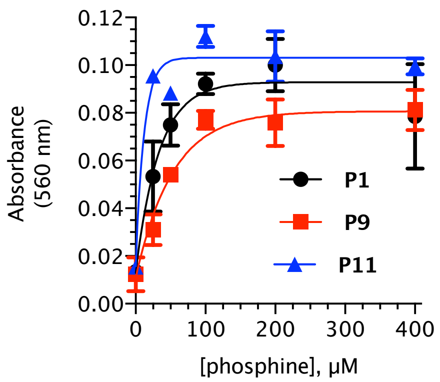

Supplementary Figure 2. Rescreening **P1**, **P9**, **P11** from phosphine screen (Supplementary Table 1). 200 μM RAE, 40 ppb Pd^{2+}, 300 μM $PhCH_2NH_2$, 0 – 400 μM phosphine, 10 mM $NaBH_4$, EtOH, 25 °C, 60 min, *n* = 1. **P9** was selected as the phosphine of choice as we could store solutions for > 6 months without degradation in the presence of 250 ppm BHT.

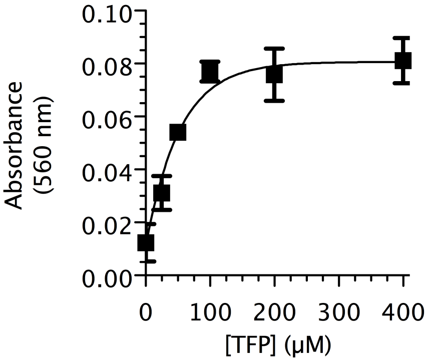

Supplementary Figure 3. Screening for optimized TFP (**P9)** concentration dependence. Conditions: 200 μM RAE, 40 ppb Pd^{2+}, 300 μM $PhCH_2NH_2$, 10 mM $NaBH_4$, 0 – 400 μM TFP, EtOH, 24 °C, 60 min. *n* = 3.

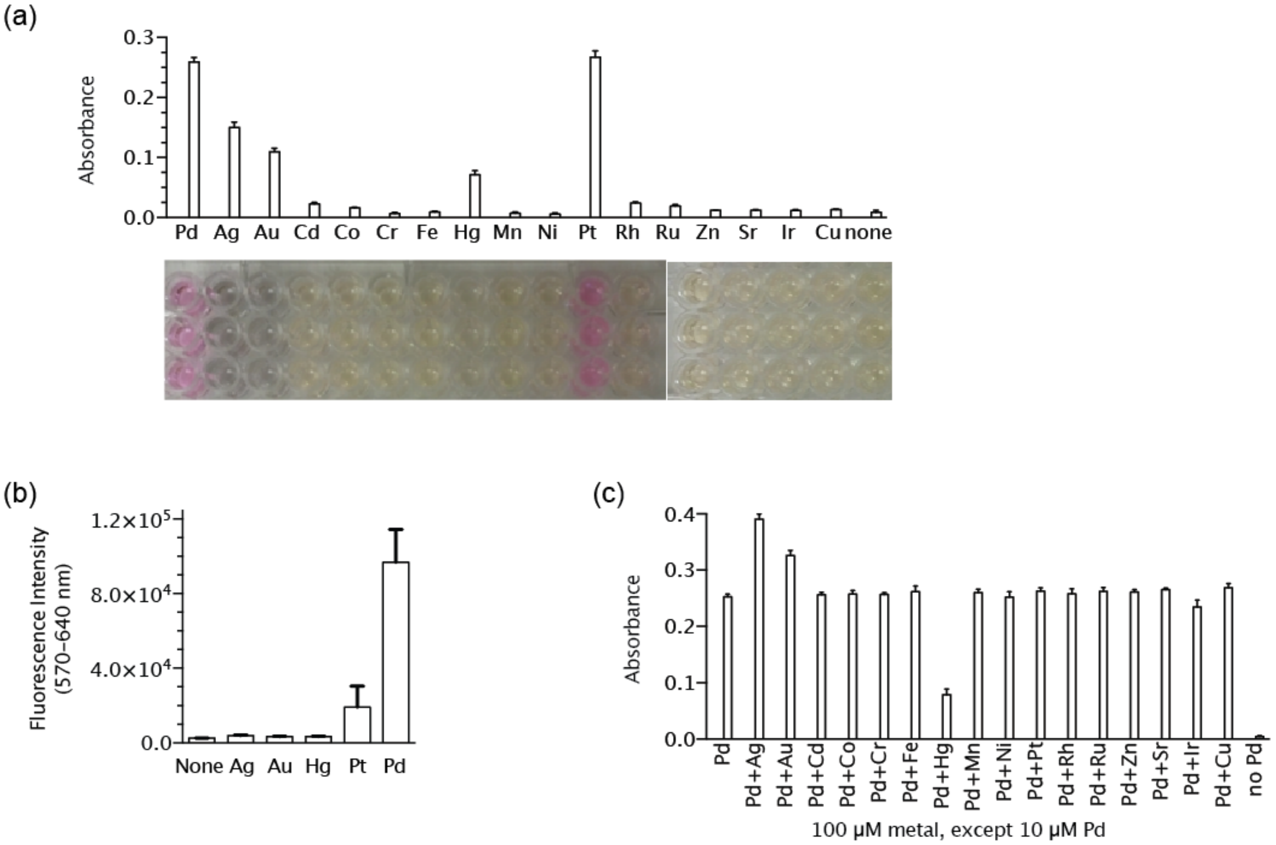

Supplementary Figure 4. Metal selectivity. (a) Metal selectivity of RAE deallylation. Conditions: 29 μM RAE, 100 μM metal except Pd^{2+} at 10 μM, 3 mM $NaBH_4$, 200 μM TFP, 800 mM NH_4OAc, EtOH, 24 °C, 60 min. *n* = 3. Higher absorbance of Ag, Hg, and Au samples was due to opacity. The photograph was taken under ambient light. (b) Fluorescence intensities vs. metal of the reaction solutions from (a). (c) Metal selectivity with mixture of Pd and other metals in a 1:10 ratio. Conditions: 29 μM RAE, 100 μM metal, 10 μM Pd^{2+}, 3 mM $NaBH_4$, 200 μM TFP, 800 mM NH_4OAc, EtOH, 24 °C, 60 min. *n* = 3.

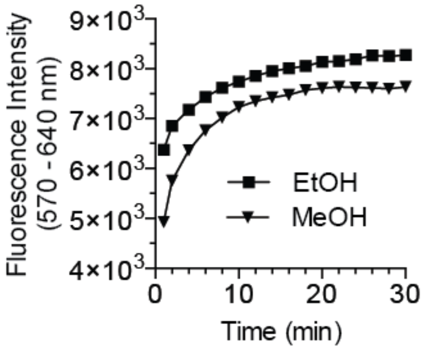

Supplementary Figure 5. Deallylation of RAE employing EtOH or MeOH as solvent. Conditions: 29 μM RAE, 200 μM TFP, 5 mM $NaBH_4$, 800 mM NH_4OAc, EtOH or MeOH, 24 °C, 30 min. n = 1.

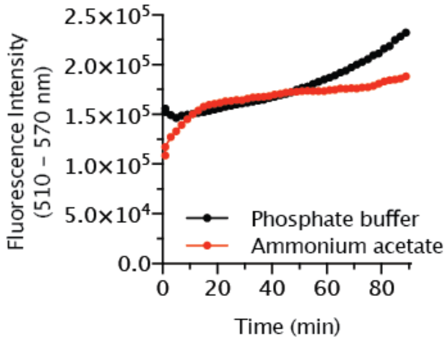

Supplementary Figure 6. Stalling APE deallylation. Conditions: 20 μM APE, 10 ppb Pd^{2+}, 80 μM TFP (**P9**), 600 mM HPO_4^{2-} or NH_4OAc, 25 °C, 90 min, 20% v/v $EtOH/H_2O$.

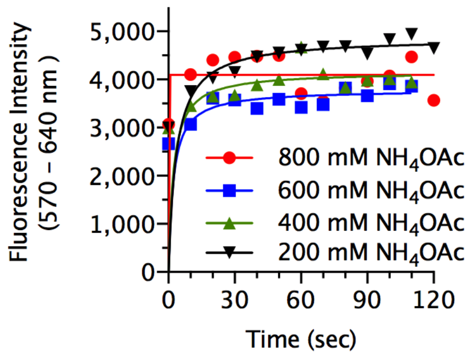

Supplementary Figure 7. Various NH_4OAc concentrations do not have an effect on reaction lifetime. Conditions: 29 μM RAE, 0.3 ppm Pd^{2+}, 200 μM TFP, 0-800 mM NH_4OAc, 0.6 mM $NaBH_4$. Samples were basified with 1 N NaOH prior to fluorescence reading.

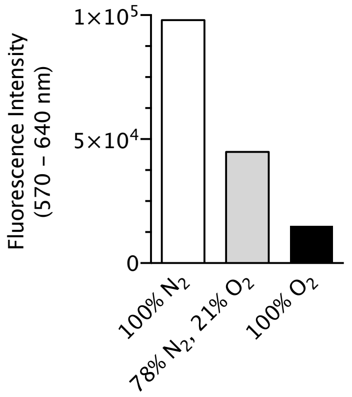

Supplementary Figure 8. Effect of O_2 on the palladium-catalyzed deallylation of RAE. Conditions: 29 μM RAE, 200 μM TFP, 1 mM $NaBH_4$, 100 ppb Pd^{2+}, 800 mM NH_4OAc, 25 °C, EtOH, 15 min, n = 1.

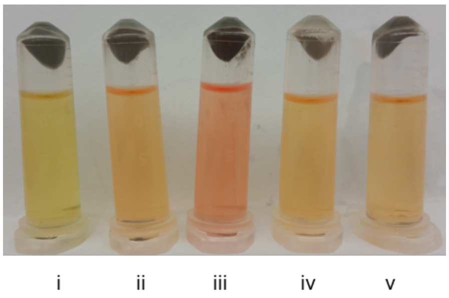

Supplementary Figure 9. Analysis of ore samples. Conditions: 29 μM RAE, 200 μM TFP, 800 mM NH_4OAc, 75 mM $NaBH_4$, 200 mg milled ore sample, EtOH, 25 °C, 20 min. Samples i, ii, and iii contain 0.030, 0.068, and 0.094% palladium by weight, respectively. Samples iv and v contain no palladium. Palladium concentrations were determined by aqua regia digestion and analysis (*1*).

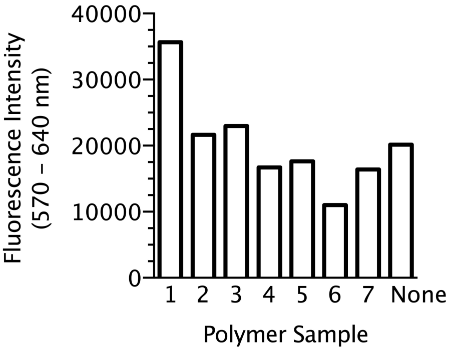

Supplementary Figure 10. Analysis of polymers after digestion with aqua regia. Conditions: 29 μM RAE, 5 ppm polymer, 200 μM TFP, 800 mM NH_4OAc, 25 mM $NaBH_4$, 25 °C, EtOH, 30 min, n = 1. Relative palladium concentrations did not correlate to amounts summarized in Supplementary Table 2.

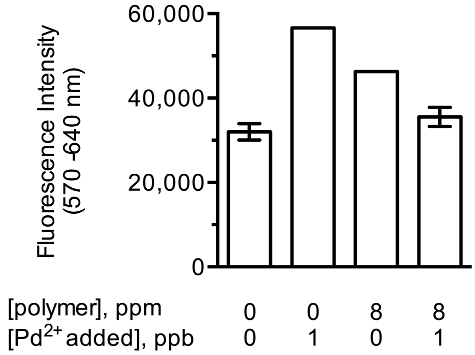

Supplementary Figure 11. Interference by polymers on analysis with RAE. Conditions: 29 µM RAE, 0 – 8 ppm polymer, 0 – 1 ppm Pd^{2+}, 200 µM TFP, 25 mM $NaBH_4$, EtOH, 24 °C, 30 min, *n* = 3.

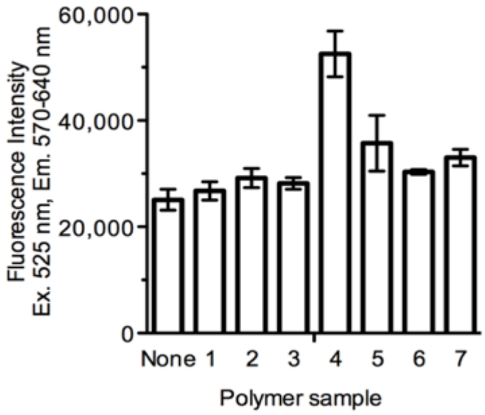

Supplementary Figure 12. Analysis of Pd impurity content in digested polymer samples. Conditions: 29 µM RAE, 16 ppm polymer, 200 µM TFP, 50 mM $NaBH_4$, 800 mM NH_4OAc, EtOH, 24 °C, 30 min, *n* = 3. Relative palladium concentrations correlated to the amount of palladium used in each sample.

Supplementary Figure 13. ^{1}H NMR spectrum of RAE: $CDCl_3$, 293K, 400 MHz

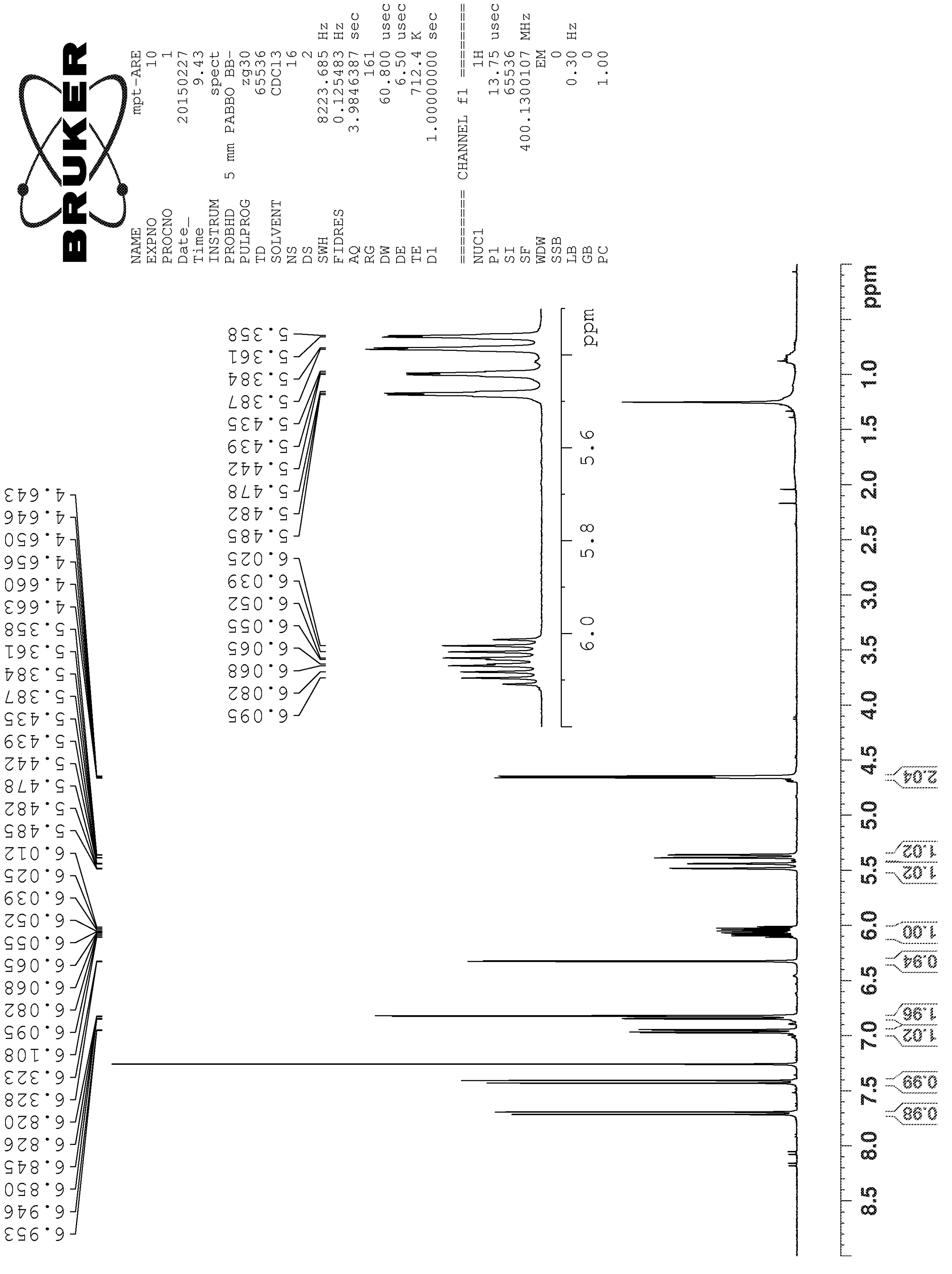

Supplementary Figure 14. ^{13}C NMR spectrum of RAE: $CDCl_3$, 293K, 75 MHz

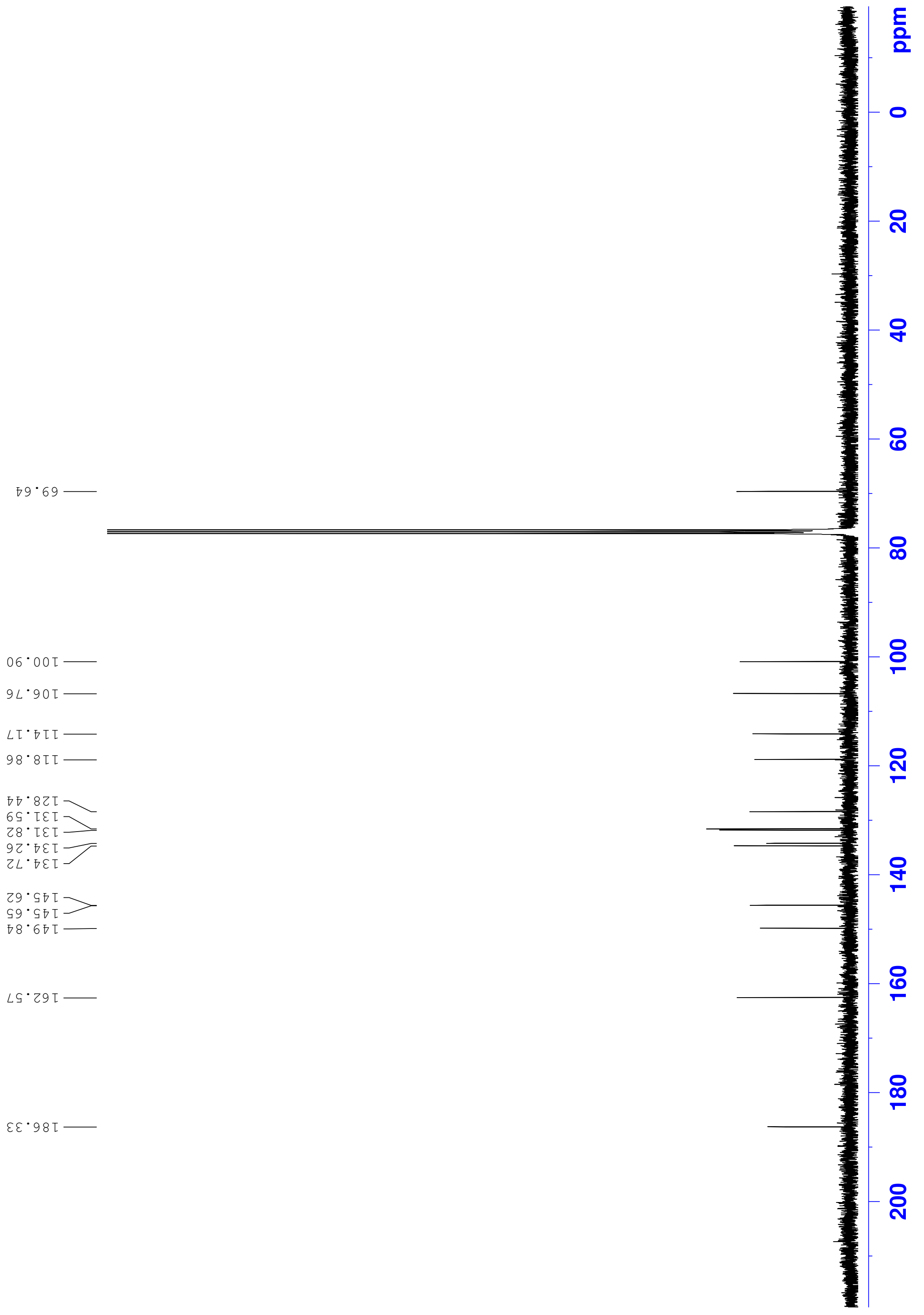

Supplementary Tables

[phosphine] in µM	P1	P2	P3	P4	P5	P6	P7	P8	P9	P10	P11	P12	P13	P14	P15
**0**	0.8	0.8	0.8	0.8	0.8	0.8	0.8	0.8	0.8	0.8	0.8	0.8	0.8	0.8	0.8
**25**	1.1	0.4	0.8	1.6	1.2	0.9	2.5	0.9	2.3	3.6	4.5	1.0	0.7	0.9	0.6
**50**	1.3	0.4	1.8	1.2	1.3	1.0	3.1	0.7	2.3	3.4	4.4	1.3	0.7	1.1	0.8
**100**	1.3	0.5	0.5	1.4	1.3	1.1	3.4	0.9	2.5	3.4	4.4	1.0	0.8	1.0	0.7
**200**	1.1	0.2	0.6	1.1	1.5	1.2	3.6	1.2	2.5	2.8	4.8	0.9	0.9	1.0	0.7
**400**	1.0	0.2	0.6	1.2	1.5	1.2	3.4	0.7	2.2	1.7	3.8	0.9	0.7	0.9	0.5

Supplementary Table 1. Raw data of the relative fluorescence intensities. Darker green indicates stronger fluorescence signals relative to 400 µM **P1**. Deallylation conditions were 200 µM RAE, 40 ppb Pd^{2+}, 300 µM $PhCH_2NH_2$, 0 – 400 µM phosphine, 10 mM $NaBH_4$, EtOH, 25 °C, 60 min, n = 1.

Polymer Sample	Polymerization Method (scale)	Mol% $Pd_2(dba)_3$	Reaction time
Sample 1	Batch (0.3 g)	3	24 h
Sample 2	FSP* (0.3 g)	1	30 min
Sample 3	FSP (0.3 g)	3	30 min
Sample 4	FSP (0.3 g)	5	30 min
Sample 5	FSP (0.3 g)	3	15 min
Sample 6	FSP (0.3 g)	3	45 min
Sample 7	FSP (10 g)	3	30 min

*FSP = Flow synthesis polymerization

Supplementary Table 2. Legend of polymers analyzed

Supplementary Notes

Supplementary Note 1: Addition of polymer solutions to a reaction cocktail containing RAE was unsuccessful (data not shown), either due to the lack of solubility of the polymer itself or due to interference from the polymer.

Supplementary Note 2: As shown in Supporting Figure 11, the presence of the digested polymer in the reaction led to inaccurate relative palladium concentrations. The subsequent analyses were performed extracting palladium from the polymer solution first.

Supplementary Methods

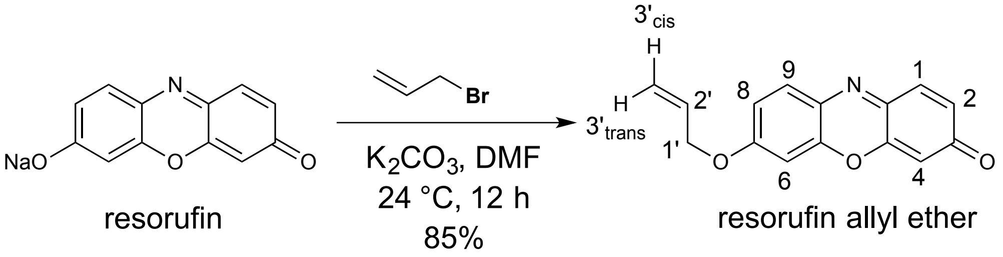

Protocol: A solution of resorufin sodium salt (200 mg, 0.850 mmol) in DMF (5 mL) was treated with K_2CO_3 (356 mg, 2.55 mmol, 3.0 equiv) followed by allyl bromide (1.4 mL, 0.94 mmol, 1.1 equiv) in DMF (1 mL) at 24 °C and stirred at the same temperature for 12 h. The reaction mixture was then quenched with water (100 mL), and the resulting mixture was filtered through a coarse fritted funnel. The resulting solid was washed with water (3 × 100 mL) and cold hexanes (100 mL). The solid was recrystallized from ethyl acetate and hexanes, affording resorufin allyl ether (181 mg, 0.71 mmol, 85% yield) as a dark red-orange solid. Data for resorufin allyl ether: Rf = 0.34 (50% ethyl acetate in hexanes); m.p.: 210.3–211.6 °C (decomp); IR: 2960, 2873, 1733 (C=O), 1466, 1369, 1151, 1031, 955.45, 862, 785, 735 cm^{-1}; ^1H NMR (400 MHz, $CDCl_3$, 293 K, Supplementary Figure 13): δ 7.71 (d, *J* = 8.8 Hz, 1H, Ar), 7.42 (d, *J* = 10.2 Hz, 1H, Ar), 6.96 (dd, *J* = 8.8, 2.4 Hz, 1H, Ar), 6.84 (dd, *J* = 10.2, 2.0 Hz, 1H, Ar), 6.82 (d, *J* = 2.4 Hz, 1 H, Ar), 6.32 (d, *J* = 2.0 Hz, 1H, Ar), 6.06 (ddt, *J* = 17.2, 10.2, 5.1 Hz, 1H, 2'-H), 5.46 (ddt, *J* = 17.2, 3.0, 1.5 Hz, 1H, 3'-H_{cis}), 5.37(ddt, *J* = 10.2, 3.0, 1.5 Hz 1 H, 3'-H_{trans}), 4.65 (ddt, *J* = 5.1, 1.5, 1.5 Hz, 2H, 1'-H). ^{13}C NMR (75 MHz, CDCl3, 293 K, Supplementary Figure 14): δ 186.3, 162.6, 149.8, 145.65, 145.62 134.7, 134.3, 131.8, 131.6, 128.4, 118.9, 114.2, 106.8, 100.9, 69.6; HRMS: (ESI+) calcd for $C_{15}H_{11}NO_3$ [M+H] = 254.0814, found 254.0798.

Determination of optimal phosphine ligand for deallylation of RAE

To separate scintillation vials was added triphenylphosphine (**P1**, Supplementary Figure 1) (26.2 mg, 0.100 mmol), 1,2-bis(diphenylphosphino)ethane (**P2**) (39.5 mg, 0.100 mmol), 1,3-bis(diphenylphosphino)propane (**P3**) (41.6 mg, 0.100 mmol), 1,1'-bis(diphenylphosphino)ferrocene (**P4**) (55.4 mg, 0.100 mmol), bis(2-diphenylphosphinophenyl)ether (**P5)** (53.8 mg, 0.100 mmol), racemic-2,2'-bis(diphenylphosphino-1,1'-binaphthalene (**P6**) (62.2 mg, 0.100 mmol), tris(2,4-di-*tert*-butylphenyl)phosphite (**P7**) (64.8 mg, 0.100 mmol), (2-biphenyl)-di-*tert*-butylphosphine (**P8**) (29.6 mg, 0.100 mmol, **8**), tri(2-furyl)phosphine (TFP) (**P9**) (23.7 mg, 0.100 mmol), 1,3,5-triazaphosphaadamantane (**P10**) (15.6 mg, 0.100 mmol), tris(4-methoxyphenyl)phosphine (**P11**) (35.2 mg, 0.100 mmol), tri-*tert*-butylphosphonium tetrafluoroborate (**P12**) (29.8 mg, 0.100 mmol), tri(*o*-tolyl)phosphine (**P13**) (30.3 mg, 0.100 mmol), tri(*p*-tolyl)phosphine (**P14**) (30.6 mg, 0.100 mmol), and tris(4-fluorophenyl)phosphine (**P15**) (31.2 mg, 0.100 mmol), and DMSO (5.0 mL, [phosphine ligand] = 20 mM). To a separate vial, an aliquot of previously prepared solutions (48 µL) were diluted in DMSO (252 µL, [phosphine] = 3.2 mM). Two-fold serial dilutions were performed to afford 3200, 1600, 800, 400, 200, and 100 µM phosphine ligand solutions in DMSO.

The reaction cocktail was prepared by mixing EtOH (23.6 mL), 800 µM RAE in EtOH (683 µL), $PhCH_2NH_2$ (621 µL), and 2.5 M $NaBH_4$ in 10 N NaOH (99 µL) at 0 °C. The resulting solution (175 µL) was distributed to each well of a black round-bottom 96-well plate. The phosphine solutions (25 µL) described above were added to each well affording final concentrations of 400, 200, 100, 50, 25, and 12.5 µM phosphine. To each well was added 1 ppm palladium standard solution in 5% TraceMetal nitric acid (10 µL, affording a final concentration of 40 ppb Pd). Fluorescence (excitation 525 nm, emission 570–640 nm) was recorded after 1, 30, and 60 min. The ratio of fluorescence was compared to 400 µM triphenylphosphine as a control and is reported. The result is shown in Supplementary Table 1.

Rescreening of active phosphine ligands

The reaction cocktail was prepared by mixing EtOH (23.6 mL), 800 µM RAE in EtOH (683 µL), $PhCH_2NH_2$ (621 µL), and 2.5 M $NaBH_4$ in 10 N NaOH (99 µL) at 0 °C. The resulting solution (175 µL) was distributed to each well of a black round-bottom 96-well plate. The phosphine solutions of **P1**, **P9**, and **P11** (25 µL) described above were added to each

well affording final concentrations of 400, 200, 100, 50, 25, and 12.5 μM phosphine. To each well was added 1 ppm palladium standard solution in 5% TraceMetal nitric acid (10 μL, affording a final concentration of 40 ppb Pd). Absorbance (560 nm) was measured after 60 min. The result is shown in Supplementary Figure 2.

Determination of optimal tri-(2-furyl)phosphine (**P9)** concentration

In a scintillation vial was added TFP (**P9**) (23.2 mg, 0.100 mmol) and DMSO containing 250 ppm BHT (5.0 mL; [TFP] = 20 mM). To a white chemically resistant plate was added this solution (192 μL) and DMSO (108 μL, [TFP] = 12.8 mM). From this solution, 2x serial dilutions in DMSO were performed to concentrations of 0.400, 0.200, 0.100, 0.0500, 0.0250, and 0 mM.

The reaction cocktail was prepared by mixing EtOH (7.6 mL), 800 μM RAE in EtOH (220 μL), benzylamine (200 μL) and 2.5 M $NaBH_4$ in 10 N NaOH (32 μL) at 0 °C. The solution (175 μL) was transferred to each reaction well in a 96-well clear, flat-bottom absorbance plate. The TFP solutions (25 μL) described above were added to each well for final concentrations of 400, 200, 100, 50, 25, and 12.5 μM. A 20 ppb Pd^{2+} standard solution in 5% TraceMetal HNO_3 (10 μL) was added to each well. The assay was performed at 23 ºC, and absorbance (560 nm) was measured after 1 and 60 min using a Modulus II Microplate Multimode Reader. The result is shown in Supplementary Figure 3.

Preparation of stock metal solutions

To separate scintillation vials were added $AgNO_3$ (425 mg, 2.50 mmol), $AuCl_3$ (759 mg g, 2.50 mmol), $CdCl_2$ (459 mg, 2.50 mmol), $CoCl_2$ (325 mg, 2.50 mmol), $CrCl_3$ (396 mg, 2.50 mmol), $FeCl_3$ (406 mg, 2.50 mmol), $HgCl_2$ (679 g, 2.501 mmol), $MnCl_2$ (315 mg, 2.50 mmol), $NiCl_2$ (324 mg, 2.50 mmol), $PtCl_2$ (665 mg, 2.50 mmol), $ZnCl_2$ (341 mg, 2.50 mmol), $Sr(NO_3)_2$ (529 mg, 2.50 mmol), $IrCl_3$ (745 mg, 2.50 mmol) and $Cu(NO_3)_2$ (336 mg, 2.50 mmol) and ultrapure H_2O (2.5 mL; [metal] = 1.0 M). To separate scintillation vials were added a metal solution (50 μL) that was prepared above, and ultrapure H_2O (4.95 mL; [metal] = 10 mM. Two serial dilutions of the metal solution (20 μL) with ultrapure water (180 μL) were carried out to afford a final concentration of 100 μM. In separate scintillation vials was added either $RuCl_3$ (10.4 mg, 50.1 μmol) or $RhCl(PPh_3)_3$ (45 mg, 50 μmol) and ultrapure H_2O (5.00 mL) To separate scintillation vials was added these solutions (100 μL) and Ultrapure (9.9 mL, [metal] = 100 μM). A 10 μM solution of $Pd(NO_3)_2$ was prepared through the serial dilution of a 100 ppm Pd^{2+} standard solution in 10% nitric acid (10 μL) with ultrapure water (990 μL).

Determination of metal selectivity for RAE

The reaction cocktail was prepared by mixing 800 mM NH_4OAc in EtOH (15 mL), 800 μM RAE in EtOH (600 μL), 3.0 M TFP (**P9)** in DMSO, stabilized by 250 ppm BHT in DMSO (1.2 mL) and 0.1 M $NaBH_4$ in 10 N NaOH (450 μL) at 0 °C. The solution (180 μL) was distributed to each well in a clear flat-bottom 96-well plate. The deallylation reaction was performed in triplicate by transferring the 100-μM metal solutions or 10 μM in the case of Pd^{2+} (20 μL) to the reaction cocktail. The well plates were incubated at 25 °C. Absorbance (560 nm) was measured 1 min and 1 h after the transfer using a Modulus II Microplate Multimode Reader. After 1 h, wells corresponding to Au, Ag, Hg, Pd, and Pt were transferred to a 96-well black fluorescence well plate and fluorescence (excitation 525 nm, emission 580–640 nm) was measured using a Modulus II Microplate Multimode Reader. The result is shown in Supplementary Figure 4a and 4b.

Determination of interference by other metals

Metal solutions were prepared as in “Metal selectivity screen”. On a black 96-well plate was added each 100 μM solution of metals in ultrapure H_2O (990 μL) that was prepared as described above and a 100 μM solution of Pd^{2+} in ultrapure H_2O (10 μL).

The reaction cocktail was prepared by mixing 800 mM NH_4OAc in EtOH (15 mL), 800 μM RAE in EtOH (600 μL), 3.0 M TFP **(P9**) in DMSO stabilized by 250 ppm BHT (1.2 mL) and 0.1 M $NaBH_4$ in 10 N NaOH (450 μL) at 0 °C. The solution (180 μL) was distributed to a clear flat-bottom 96-well plate. The deallylation reaction was performed in triplicate by transferring the metal solutions described above (20 μL) to the reaction cocktail on the plate. The well plates were incubated at 25 °C. Absorbance (560 nm) was measured 1 min and 1 h after the transfer using a Modulus II Microplate Multimode Reader. The result is shown in Supplementary Figure 4c.

Testing effect of alcohol solvent on deallylation

To a 2-mL Eppendorf tube was added 800 mM NH_4OAc in ROH (1 mL, R = Me, Et). To this solution was added 3 mM TFP stabilized by 250 ppm BHT in DMSO (80 µL) and 800 µM RAE in EtOH (40 µL). To the resulting solutions was added either 0 or 500 ppb Pd^{2+} in 5% HNO_3 (20 µL) and 0.1 M $NaBH_4$ in 10 N NaOH (50 µL). The solutions (200 µL) were transferred to a black 96-well fluorescence plate and fluorescence (excitation 525 nm, emission 570–640 nm) was recorded every 1 min for 30 min. The result is shown in Supplementary Figure 5.

Testing allyl Pittsburgh Green ether (APE) for autonomous stalling with NH_4OAc

The acetate-containing reaction cocktail was prepared by mixing NH_4OAc (440 mg, 5.71 mmol) with EtOH (2 mL) and ultrapure H_2O (7.5 mL), 800 µM APE in DMSO (250 µL), and 3.2 mM TFP in DMSO stabilized with 250 ppm BHT (250 µL). In a separate vial, the phosphate-containing reaction cocktail was prepared by mixing 1.2 M potassium phosphate pH 7 buffer (5 mL), EtOH (2 mL), ultrapure H_2O (2.5 mL), 800 µM **1a** in DMSO (250 µL), and 3.2 mM TFP (**P9**) in DMSO stabilized with 250 ppm BHT (250 µL).

The reaction cocktail (1 mL) was added to 2-mL Eppendorf tubes. To half of the samples for each buffer was added 5% TraceMetal HNO_3 (20 µL) as a control. To the other half of the samples was added 50 ppb Pd^{2+} in 5% TraceMetal HNO_3 (20 µL). To all samples was added 0.5 M $NaBH_4$ in 10 N NaOH (20 µL). The samples were mixed and added (200 µL) to a 96-well black fluorescence well plate. Fluorescence (excitation 490 nm, emission 510–570 nm) was measured every 2 min for 60 min using a Modulus II Microplate Multimode Reader. The result is shown in Supplementary Figure 6.

Deallylation under inert atmosphere

Three separate round-bottom flasks were vacuumed and sealed with a rubber stopper. Either a balloon of N_2, O_2, or air (78% N_2, 21% O_2) was then attached with a needle. A scintillation vial was treated with 800 mM NH_4OAc in EtOH (9 mL), 3 mM TFP in DMSO stabilized by 250 ppm BHT (720 µL), and 800 µM RAE in EtOH (360 µL). Aliquots (2 mL) of this solution were added to each flask followed by 1 ppm Pd^{2+} in 5% HNO_3 (100 µL) and 40 mM $NaBH_4$ in 10 N NaOH (50 µL) via syringe. After 15 min, aliquots (200 µL) of each solution were transferred to a black 96-well plate, and fluorescence (excitation 525 nm, emission 570–640 nm) was measured using a Modulus II Microplate Multimode Reader. The result is shown in Supplementary Figure 8.

Analysis of ores

Ore samples used were provided by Stillwater Mining Co. and were previously analyzed for palladium content using APE following aqua regia digestion and by solid-state palladium extraction Initial attempts to directly analyze the samples with RAE failed, likely due to difficulty in palladium extraction from the rock samples in the short reaction time. Whereas our previous method with APE could extract palladium and the reaction would run indefinitely, our new method stalls before a signal could be recovered and before significant palladium was extracted. To alleviate this with the single cocktail, we extracted palladium using TFP in DMSO followed by the addition of RAE and NH_4OAc-containing EtOH. $NaBH_4$ was subsequently added to the samples.

To 2-mL Eppendorf tubes were added ore samples (75 mg, Note: These samples contained ranges of 0 to 0.068% Pd by weight as determined by previous analysis using APE (*1*) and 3 mM TFP in DMSO stabilized by 250 ppm BHT (145 µL). Samples were sonicated for 60 s. To a scintillation vial was added 800 mM NH_4OAc in EtOH (6.48 mL) and 800 µM RAE in EtOH (259 µL). This solution (1.86 mL) was added to each Eppendorf tube containing ore samples. To each sample was added 2.5 M $NaBH_4$ in 1 N NaOH (20 µL). The samples were incubated at 25 °C for 5 min. An additional 2.5 M $NaBH_4$ in 1 N NaOH (20 µL) was added and again the samples were incubated for 5 min. To each sample was again added 2.5 M $NaBH_4$ in 1 N NaOH (20 µL, 75 mM $NaBH_4$ final) and the samples were incubated for 5 min, centrifuged using a Galaxy II Mini benchtop centrifuge for 20 s, and the resulting slurry was recorded with a photograph obtained under ambient light. The result is shown in Supplementary Figure 9.

Analysis of polymers by colorimetric detection

We applied the colorimetric assay to the detection of residual Pd impurities in organic polymers, a problem of increasing concern in the specialty chemical arena, where such impurities can compromise the performance characteristics of organic polymers prepared by either batch preparation or flow synthesis polymerization. Various polymer samples, prepared by either batch or flow synthesis with varying amounts of palladium catalyst by the Krebs group (Supplementary Table 2), were dissolved in either $CHCl_3$ or PhMe and analyzed using RAE.

Analysis of polymers prepared by palladium catalysis

Preparation of polymer solutions

Solutions of polymers provided by the Krebs laboratory (Technical University of Denmark) were dissolved in either toluene ("Sample 2") or $CHCl_3$ ("Sample 1,3,4,5,6,7") to afford concentrations of 1,000 µg/mL (1,000-ppm) of polymer.

Analysis of polymers by aqua regia digestion

To separate 2-dram vials were added polymer solution (400 µL), placed under a stream of air at 25 °C and evaporated to dryness. To these vials was added freshly prepared aqua regia (100 µL) and the samples were allowed to digest for 16 h at 25 °C. To each vial was added ultrapure H_2O (900 µL).

Reaction cocktail described in "General protocol for deallylation of RAE" (1 mL) was added to 2-mL Eppendorf tubes and to this solution was added either 10% aqua regia or polymer suspension in 10% aqua regia (20 µL, [polymer] = 5 ppm) and 2.5 M $NaBH_4$ in 10 N NaOH (20 µL). Each solution (200 µL) was transferred to a black 96-well fluorescence plate and the fluorescence (excitation 525 nm, emission 570–640 nm) was measured after 30 min. The result is shown in Supplementary Figure 10. The observed result did not match the expected palladium concentration shown in Supporting Table 2.

Determination of interference by polymers

To separate 2-dram vials was added 1000 ppm "Sample 4" in $CHCl_3$ (400 µL). Vials were placed under a stream of air at 25 °C and evaporated to dryness. To these vials was added freshly prepared aqua regia (100 µL) and the samples were allowed to digest for 16 h at 25 °C. To each vial was added ultrapure H_2O (900 µL).

Reaction cocktail described in "General protocol for deallylation of RAE" (1 mL) was added to 2-mL Eppendorf tubes and to this solution was added either 10% aqua regia or polymer suspension in 10% aqua regia (20 µL, [polymer] = 8 ppm), to half of the samples was added H_2O, to the other half was added 50 ppb Pd^{2+} in H_2O (20 µL), and 2.5 M $NaBH_4$ in 10 N NaOH (20 µL). Each solution (200 µL) was transferred to a black 96-well fluorescence plate and the fluorescence (excitation 525 nm, emission 570–640 nm) was measured every 2 min for 30 min. The result is shown in Supplementary Figure 11

To alleviate the problem, we turned to acid digestion, removing aliquots of the polymer solutions, evaporating them to dryness, and resuspending them in 5% HNO_3. Heating the samples affected a rapid extraction of palladium, and subsequent analysis using RAE revealed relative palladium concentrations in each polymer. The highest palladium-containing polymer was subjected to further analysis, highlighting the use of RAE as a colorimetric chemodosimeter for analysis of digested polymers.

Heat-assisted polymer analysis

To separate 2-dram vials were added 1000 ppm polymer solutions in toluene ("Sample 2") or $CHCl_3$ ("Samples 1,3,4,5,6,7") (400 µL). The vials were placed under a stream of air at 25 °C and evaporated to dryness. To each vial was added 5% TraceMetal HNO_3 (500 µL) and the resulting suspensions (800 ppm polymer) were loosely sealed with threaded tape and screw caps and incubated in a 70 °C water bath for 3 h.

Reaction cocktail described in "General protocol for deallylation of RAE" (1 mL) was added to 2-mL Eppendorf tubes and to each vial was added either 5% TraceMetal HNO_3 or polymer suspension in 5% HNO_3 (20 µL, [polymer] = 16 ppm) and 2.5 M $NaBH_4$ in 10 N NaOH (20 µL). Each solution (200 µL) was transferred to a black 96-well

fluorescence plate and the fluorescence (excitation 525 nm, emission 570–640 nm) was measured after 30 min. The result is shown in Supplementary Figure 12.

<u>Inhibition of horseradish peroxidase</u>

Amplex Red (a.k.a. Ampliflu Red, 10-acetyl-3,7-dihydroxyphenoxazine) was purchased from Life Technologies (Catalog no. A22188) and was stored at -20 °C in single use ampules as provided. To a scintillation vial was added horseradish peroxidase (3.3 mg 303 U/mg) and 1× PBS (10 mL, 10 U/mL final concentration). The solution was separated into single use 1 mL aliquots and stored at -20 °C. To a 2-dram vial was added $PhB(OH)_2$ (15, 30, 150 mg; 0.12, 0.14, 1.2 mmol) and 1× PBS (4.96 mL) to afford stock solutions. To each solution of $PhB(OH)_2$ was added either H_2O or 6.36 mM H_2O_2 in H_2O (40 μL) and the solutions were incubated at 24 °C for 10 min. While the solutions were incubating, to a single ampule of Amplex Red (154 μg) was added DMSO (60 μL). The reaction cocktail was prepared by combining 10 mM Amplex Red in DMSO (50 μL), 10 U/mL HRP in 1× PBS (100 μL), and 1× PBS (4.85 mL). Aliquots of the reaction cocktail (50 μL) were transferred to a 96-well black fluorescence plate. To each well was added either 1× PBS with or without 50 μM H_2O_2 containing 50, 100, or 500 μM $PhB(OH)_2$. The plate was incubated at 25 °C and fluorescence intensity (excitation 525 nm, emission 570–640 nm) was measured after 30 min. The result is shown in Figure 5b.

<u>Restarting a stopped HRP reaction</u>

A 20 mM stock solution of $PhB(OH)_2$ was prepared by adding $PhB(OH)_2$ (24 mg, 0.20 mmol) to 1× PBS (10 mL). This solution was divided into aliquots (1.5 mL) and to each was added either H_2O or 1.05 M H_2O_2 in H_2O (30 μL). A reaction cocktail was prepared by combining 10 mM Amplex Red in DMSO (25 μL), 10 U/mL horseradish peroxidase (25 μL), and 1× PBS (2.5 mL). Aliquots of the reaction cocktail (50 μL) were transferred to a 96-well black fluorescence plate and to each well was added either 1× PBS with or without 10 μM H_2O_2 containing 0 or 10 mM $PhB(OH)_2$ (50 μL). Fluorescence intensity (excitation 525 nm, emission 570–640 nm) was measured every 2 min for 10 min. To each well was again added either 1× PBS with or without 10 μM H_2O_2 containing 0 or 10 mM $PhB(OH)_2$ (50 μL). Fluorescence intensity (excitation 525 nm, emission 570–640 nm) was measured every 2 min for 10 min. Data presented in Figure 6c are normalized to account for the dilution from adding the second aliquot (50 μL) to the solutions on the plate (100 μL) to afford a final volume of 150 μL. Fluorescence intensities are scaled to 1.5x observed values to account for this. The result is shown in Figure 5c.

<u>Observed saturation in restarting a stopped HRP reaction</u>

A 40 mM stock solution of $PhB(OH)_2$ was prepared by adding $PhB(OH)_2$ (48 mg, 0.40 mmol) to 1× PBS (10 mL). This solution was divided into aliquots (1.5 mL) and to each was added either H_2O or 1.05 M H_2O_2 in H_2O (30 μL). A reaction cocktail was prepared by combining 10 mM Amplex Red in DMSO (25 μL), 10 U/mL horseradish peroxidase (250 μL), and 1× PBS (2.25 mL). Aliquots of the reaction cocktail (50 μL) were transferred to a 96-well black fluorescence plate and to each well was added either 1× PBS with or without 10 μM H_2O_2 containing 0 or 20 mM $PhB(OH)_2$ (50 μL). Fluorescence intensity (excitation 525 nm, emission 570–640 nm) was measured every 2 min for 10 min. To separate scintillation vials was added 0 or 10 mM $PhB(OH)_2$ in 1× PBS (1.5 mL) and either H_2O (60 μL) or 1.05 M H_2O_2 (60 μL), affording new stock solutions. To each well was added the new stock solution, either 1× PBS with or without 20 μM H_2O_2 containing 0 or 20 mM $PhB(OH)_2$ (50 μL). Fluorescence intensity (excitation 525 nm, emission 570–640 nm) was measured every 2 min for 10 min. Data presented in Figure 6d are normalized to account for the dilution from adding the second aliquot (50 μL) to the solutions on the plate (100 μL) to afford a final volume of 150 μL. Fluorescence intensities are scaled to 1.5x observed value to account for this. The result is shown in Figure 5d.

Supplementary References

1. J. M. Williams, K. Koide, A high-throughput method to detect palladium in ores. *Ind. Eng. Chem. Res.* **52**, 8612–8615 (2013).
